# Identification of Genomic Loci and Candidate Genes Related to Seed Tocopherol Content in Soybean

**DOI:** 10.3390/plants11131703

**Published:** 2022-06-27

**Authors:** Suprio Ghosh, Shengrui Zhang, Muhammad Azam, Kwadwo Gyapong Agyenim-Boateng, Jie Qi, Yue Feng, Yecheng Li, Jing Li, Bin Li, Junming Sun

**Affiliations:** 1 The National Engineering Research Center of Crop Molecular Breeding, MARA Key Laboratory of Soybean Biology (Beijing), Institute of Crop Sciences, Chinese Academy of Agricultural Sciences, 12 Zhongguancun South Street, Beijing 100081, China; fssuprio@gmail.com (S.G.); zhangshengrui@caas.cn (S.Z.); azaamuaf@gmail.com (M.A.); k.g.agyenim.boateng@gmail.com (K.G.A.-B.); qjycyz@gmail.com (J.Q.); 82101179104@caas.cn (Y.F.); 15828459537@163.com (Y.L.); lijing02@caas.cn (J.L.); 2 Bangladesh Agricultural Research Institute, Gazipur 1701, Bangladesh

**Keywords:** soybean (*Glycine max* L. Merrill), tocopherols, next-generation sequencing (NGS), bulk segregant analysis (BSA), SNP-index, candidate genes

## Abstract

Soybean seeds are primary sources of natural tocopherols used by the food and pharmaceutical industries, owing to their beneficial impacts on human health. Selection for higher tocopherol contents in seeds along with other desirable traits is an important goal in soybean breeding. In order to identify the genomic loci and candidate genes controlling tocopherol content in soybean seeds, the bulked-segregant analysis technique was performed using a natural population of soybean consisting of 1525 accessions. We constructed the bulked-segregant analysis based on 98 soybean accessions that showed extreme phenotypic variation for the target trait, consisting of 49 accessions with extremely-high and 49 accessions with extremely-low tocopherol content. A total of 144 variant sites and 109 predicted genes related to tocopherol content were identified, in which a total of 83 genes were annotated by the gene ontology functions. Furthermore, 13 enriched terms (*p* < 0.05) were detected, with four of them found to be highly enriched: response to lipid, response to abscisic acid, transition metal ion transmembrane transporter activity, and double-stranded DNA binding. Especially, six candidate genes were detected at 41.8–41.9 Mb genomic hotspots on chromosome 5 based on ANNOtate VARiation analysis. Among the genes, only *Glyma.05G243400* carried a non-synonymous mutation that encodes a “translation elongation factor EF1A or initiation factor IF2gamma family protein” was identified. The haplotype analysis confirmed that *Glyma.05G243400* exhibited highly significant variations in terms of tocopherol content across multiple experimental locations, suggesting that it can be the key candidate gene regulating soybean seed tocopherols. The present findings provide novel gene resources related to seed tocopherols for further validation by genome editing, functional characterization, and genetic improvement targeting enhanced tocopherol composition in soybean molecular breeding.

## 1. Introduction

Vitamin E (VE) is an important health-promoting element in oilseed crops and is highly esteemed in foods, medicines, cosmetics, and animal feed [1,2]. VE is composed of two lipophilic or lipid-soluble compounds of tocopherol and tocotrienol, collectively known as tocochromanols, which are endowed with potent antioxidant or reactive oxygen species (ROS) scavenging properties [3,4,5,6]. In higher plants, tocopherol, which is the most commonly known form of VE, participates in intracellular signaling, cell membrane stability, as well as oil and protein quality improvement [7,8]. Tocopherol also plays several essential roles in the maintenance of seed shelf life, seed vigor, and plant resilience under stress [4]. Tocopherol is synthesized only in photosynthetic organisms, with the biosynthesis processes occurring through three key biochemical pathways [6]. The first two pathways take place through the condensation of two precursor compounds: (1) a polar chromanol group-homogentisate which is synthesized via the cytosolic shikimate (SK) pathway, and (2) an isoprenoid phytyl chain-phytyl diphosphate synthesized through the plastidial methylerythritol phosphate (MEP) pathway [5,9,10]. The two pathways, SK and MEP, occur upstream of the third biosynthesis pathway (the tocopherol-core pathway) and are regulated by several biochemical steps or enzymes that provide metabolic influx into the tocopherol-core pathway [7]. Besides being precursor pathways for the biosynthesis of tocopherol, MEP and SK are also connected to other metabolic pathways, such as carotenoids, chlorophylls, gibberellins, phylloquinone, and plastoquinone [11]. Tocopherol exists in four different isoforms designated as α-tocopherol, β-tocopherol, γ-tocopherol, and δ-tocopherol and differ from one another by the number and position of methyl groups on the chromanol head group [2,5,6]. Tocopherol is an important nutritional compound in many oilseed crops, including soybean.

Soybean is one of the globally most significant leguminous crop species with an ever-increasing demand for its grains and processed products [12]. Soybeans are recognized as a storehouse of nutrients. The diversity of the beneficial health-promoting compounds in the grains makes soybean one of the most important oilseed crops across the world [13]. Soybean seed contains approximately 21% oil and 40% protein, in addition to several important health-promoting nutritional constituents such as isoflavones and vitamins [14,15]. Tocopherols are present in relatively high concentrations in soybeans. In soybean and other oilseed crop species, tocopherol is extracted together with oil [16] and contributes significantly to the oil quality. Tocopherol composition is inherited as a quantitative trait and thus can be genetically dissected [17]. Consequently, genetic improvement of seed composition attributes, including tocopherol composition in soybean, has become a major research goal in recent times. Thus far, a plethora of genes have been identified that mediate the biosynthesis processes leading to the formation of tocopherol isoforms, yet many of these genes have not been fully unraveled in soybean.

Next-generation sequencing (NGS) is a high-throughput molecular technology and has been used to identify several informative single nucleotide polymorphism (SNP) markers in a cost-effective and rapid fashion in diverse species. NGS techniques continue to provide new ways that can accelerate the genetic analysis of traits, such as candidate gene identification. One of the major applications of NGS in recent times involves the use of bulked segregant analysis (BSA) coupled with whole-genome resequencing (WGR) aimed at candidate gene identification and fine-mapping of causal polymorphisms. Thus, the bulked segregant analysis, an NGS technique popularly known as BSA-seq, has been applied in the genetic analysis since the early 1990s to elucidate gene conditioning phenotypic variations in organisms [18,19]. The advent of BSA-seq has reduced the application of the classical positional cloning methodology largely due to its endowed characteristic high throughput, the reduced genotyping workload, and the time-saving genotyping process [19,20]. In addition, unlike the traditional positional cloning method, BSA-seq pipelines are diverse and hence more amenable to different mapping populations that have significant phenotypic differences for a target trait, encompassing mutant libraries, segregating populations derived from systematic genetic crosses, as well as natural populations or germplasm panel [21,22,23,24,25,26]. In principle, to identify a candidate gene using BSA-seq, two separate DNA bulk samples derived from individuals exhibiting extremely contrasting differences for the target trait are composed, sequenced, and subsequently analyzed. Over the years, using the BSA-seq strategy, genomic loci or causal genes regulating vital traits have been reported in different crop species [27,28]. Further, those genes or loci have been utilized as targets for functional gene validation by genetic and genome engineering.

Currently, molecular breeding techniques have been ubiquitously applied in soybean research and have become a fundamental means of increasing the quantitative and qualitative yields of the crop [29]. Recent research has seen the frequent application of the BSA-seq technique in soybean genetic and molecular studies, with a suite of candidate genes currently available for molecular breeding of the crop. However, many previous studies involving candidate gene detection focused on the use of mutant libraries and segregating populations [16,19], with only a few reported works that targeted variations present in natural mapping populations [22]. Additionally, several previous reports on candidate gene identification in the crop have been designed to target plant shoot architecture-related traits, with only minimal work conducted on nutritional composition attributes. The past few years have seen a growing research interest in mapping causal genes and discovering the genetic mechanisms conditioning nutritional composition traits in crops [1,2,6,30,31]. For instance, based on recombinant inbred lines (RILs), QTLs associated with VE composition in soybean have been identified in multiple locations [2,16]. For the present experimental investigation, we used a natural population of global soybean collections to construct two separate DNA pools for BSA-seq and detected some novel candidate genes associated with seed tocopherols in soybean. The results of the present study can lay a foundation for further research in functional gene validation and provide the novel genotypes with conferred increased seed tocopherol content for soybean breeding.

## 2. Results

### 2.1. Pooling of the Accessions with Extreme Tocopherol Content

Based on the results of the tocopherol profiling analysis, genomic DNA samples of 49 accessions produced extremely high tocopherol content (mean: 292.95 μg·g^−1^, ranging from 276.151 to 310.167 μg·g^−1^) (Table S1), and genomic DNA samples of another 49 accessions that yielded extremely low tocopherol content (mean: 165.83 μg·g^−1^, ranging from 122.297 to 187.529 μg·g^−1^) (Table S2) among the 1525 natural population (Table S3) were sampled and used to construct two DNA pools (High-Tocopherol Pool and Low-Tocopherol Pool) (Figure S1). The accession’s phenotypic data of tocopherol content distribution indicated that the mapping population was suitable for conducting a BSA-seq study.

### 2.2. Whole-Genome Resequencing

The two DNA bulks (high and low tocopherol content) were used to perform high-throughput BSA sequencing using an Illumina HiSeq platform (NovaSeq 6000; Illumina, San Diego, CA, United States) to identify the genomic variation between the two pools and, consequently, detect the putative candidate genomic loci and genes associated with tocopherols in soybean. The sequencing data generated 53.07 Gb (53,070,136,800 bp) and 57.65 Gb (57,645,759,600 bp) of high-quality clean reads of the high (VE-High) and low (VE-Low) bulks, respectively. Table 1 and Table 2 provide detailed information on the sequencing data quality. The clean reads were aligned and mapped to the soybean Williams 82 reference genome (Wm82.a2.v1), and variant calling was performed with Genome Analysis Toolkit (GATK) software. High-quality sequencing data were obtained after filtering with an average Q20 and Q30 of 97.66% and 93.37%, respectively, in the VE-Low bulk (Table 1). The Q20 and Q30 values were 97.41% and 92.77%, respectively, in the VE-High bulk. The guanine-cytosine (GC) content of the high and low bulks was 36.52% and 36.25%, respectively (Table 1). The mapping results obtained after sequencing alignment, including average sequencing depth of the reference genome and the coverage of 1× (coverage of at least one base), was normal and thus used for further analyses such as detecting variation (SNP and InDel) between the two bulks. Following the alignment to the reference genome, the mapped rate of the two samples was 98.83% for the VE-High bulk and 98.79% for the VE-Low bulk (Table 2). The average sequencing depth of each of the two bulks was 41.5× (VE-High bulk) and 44.57× (VE-Low bulk) (Table 2). Therefore, both the quality and quantity of the data guaranteed the success of the library construction.

### 2.3. Genomic Variation (SNP/InDel) Detection and Annotation

Single-nucleotide polymorphisms (SNPs) are nucleotide variations in the genome, such as conversions and reversal of single bases. Insertion-deletions (InDels), on the other hand, are the insertions and missing genomic sequences of small DNA fragments. Following the alignment of reads to the soybean reference genome version 2.0 (Wm82.a2.v1), variant (SNPs and Indels) calling for the two bulks was performed. Variant detection was achieved with the SAMtools program [32]. Poorly supported SNPs were then filtered out. The physical positions of SNP were aligned, and the ANNOVAR algorithm was used to annotate the variants. Overall, a total of 3,559,368 SNPs and 587,007 InDels variants (Table 3) were detected between the two bulks. The majority of the genomic variants occurred in the intergenic region (Table 3 and Figure S2). Based on gene models previously reported to be linked to tocopherol biosynthesis in Arabidopsis, a total of 109 candidate genes carrying 144 genomic variants were predicted in our BSA-seq that are associated with soybean tocopherol content (Table S4).

### 2.4. Identification of Candidate Genes

**Figure 1:**
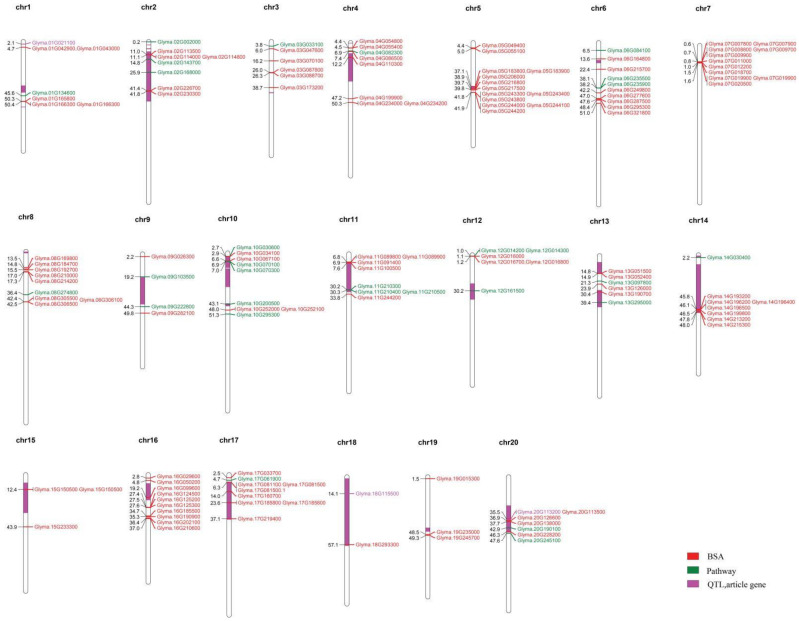
The positions of tocopherol-related loci and corresponding candidate genes on different chromosomes of soybean.

In this study, a total of 109 candidate genes (Figure 1) consisting of 144 variant sites were detected, of which 83 and 61 contained SNPs and InDels, respectively (Table S4). Among these, 13 genes were identified in the candidate region, out of which there were 10 non-synonymous, 1 stop gain, and 2 frameshift variants (Table 4). Based on gene annotation, genes in the exonic region containing non-synonymous, stop-gain or stop-loss and frameshift variations were preferentially selected as candidate genes.

Among the candidate genes detected in this study, Glyma.08G184700 and Glyma.15G233300 had frameshift deletion mutation effects. Glyma.08G184700 and Glyma.15G233300 carried genomic variants of Chr08_14819413 and Chr15_43894622, respectively (Table 4). Glyma.08G184700 (ortholog of AT5G24750) was annotated as UDP-Glycosyltransferase superfamily protein while Glyma.15G233300 (ortholog of AT1G55020) encodes a lipoxygenase 1. These two annotations (UDP-Glycosyltransferase superfamily protein and lipoxygenase 1) have been reported and may have a functional role in regulating the tocopherol composition of soybean. Further, we constructed a physical map of all the 109 putative genes that were found distributed across the entire 20 chromosomes of the soybean (Figure 1).

### 2.5. Gene Identification by Sliding Window Analysis

**Figure 2:**
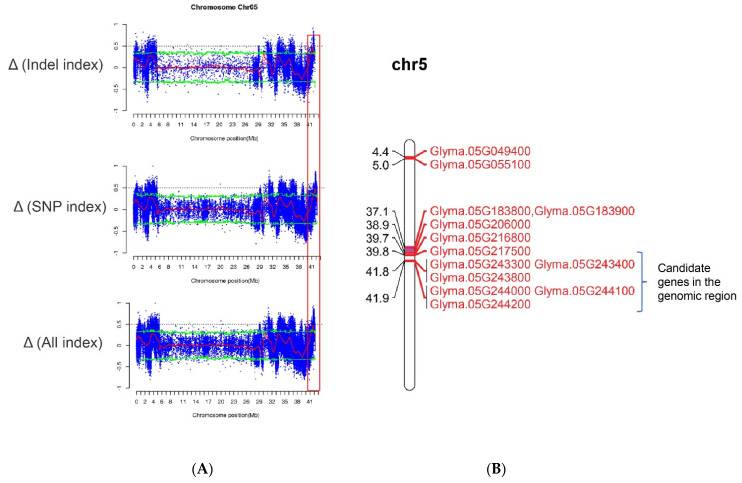
Variant association analyses for identification of the candidate regions related to tocopherol content in soybean based on BSA-seq of a panel of soybean accessions. (**A**) Visualization of the ∆(InDel index) and ∆(SNP−index) plots with statistical confidence intervals (*p <* 0.05). The dotted ash line represents SNP/InDel indices. The blue dots and red line represent ∆(SNP−index) and the sliding window average of ∆(SNP/InDel−index) calculated based on a 2 Mb interval with a 10kb sliding window. The green dotted line shows the association threshold value (0.90). (**B**) Candidate genes in the genomic locus (41.8 and 41.9) on chromosome 5.

**Figure 3:**
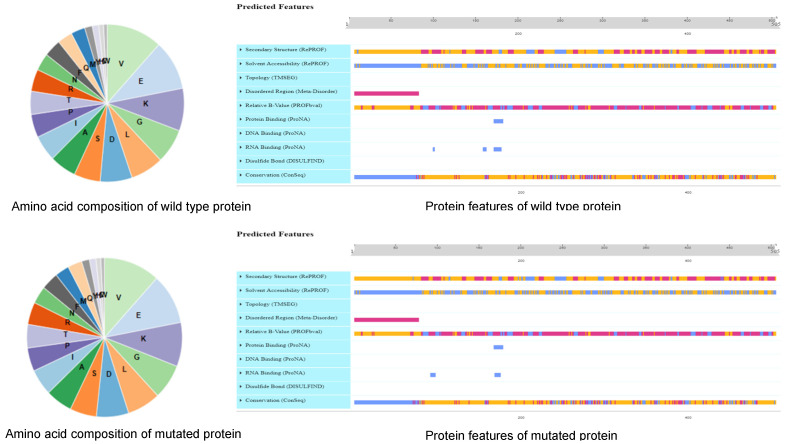
The protein structure (motifs) variants of Glyma.05G243400 between reference (WT) and mutated proteins using the PredictProtein software: https://predictprotein.org (accessed on 1 June 2022).

**Figure 4:**
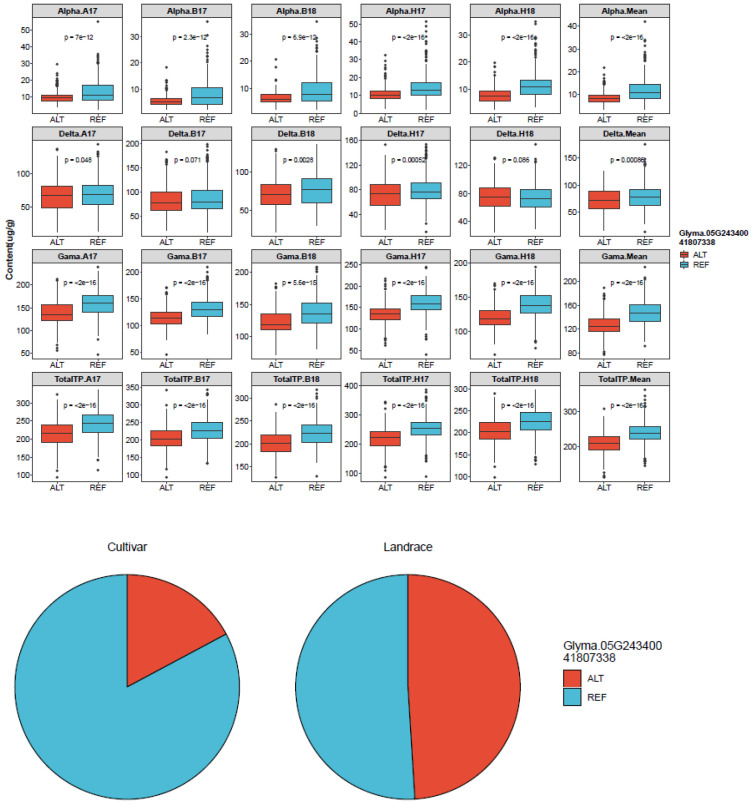
Haplotype analysis of Glyma.05G243400 in the natural soybean population. Here, REF represents the reference allele (allele with more counts in the dataset and has the same identity as that of the reference genome), while ALT represents the alternate allele count (allele not already represented by the REF). A17, Anhui 2017; B17, Beijing 2017; B18, Beijing 2018; H17, Hainan 2017; H18, Hainan 2018.

The SNP/InDel index is the most commonly used method in BSA-seq analysis. In this analysis, the reference genome base (REF: the nucleotide base with the same identity as that of the reference genome) and corresponding alternative (ALT) base are first determined for each SNP. Subsequently, the ALT read is divided by the total read (i.e., REF read + ALT read) in the bulk to give an SNP/InDel index, also known as the allele frequency. A delta SNP/InDel index or Δ(SNP/InDel-index) is finally computed as the difference of the SNP/InDel indices between the two bulks, where a higher Δ(SNP/InDel index) indicates that it is more likely that the SNP is linked to a gene conditioning the target trait. By examining the Δ(SNP/InDel-index) plots, the peak regions exceeding the threshold value were defined as putative candidate regions or regions with the fitted values greater than the standard deviations above the genome-wide media. Only peak regions that were highly consistent across the SNPs and InDels plots were considered putative genes associated with tocopherols. Based on a physical map constructed for all the 109 putative genes (Figure 1) and the associated genomic region analysis of the highest peak, we detected a genomic interval of 0.1 Mb (41.8 and 41.9 Mb) likely to harbor the gene or genes regulating tocopherol production in soybean on chromosome 5 (Figure 2). The peak regions were highly consistent across the SNPs/InDels index, and Δ(SNP/InDel-index) plots at a 95% significant level (Figure 2). Among the genes detected on chromosome 5, only *Glyma.05G243400* contained non-synonymous mutation encoding “translation elongation factor EF1A or initiation factor IF2gamma family protein” (Table S5 and Figure 3). Analysis of the haplotype variation also confirmed that *Glyma.05G243400* exhibited highly significant variations in terms of tocopherol content across the experimental locations (Figure 4).

### 2.6. GO Enrichment Analysis of Candidate Genes

The gene ontology (GO) database has a standard biological functions annotation system to produce functional annotation terms of genes and gene products. In this study, a total of 109 candidate genes (Figure 1) carrying 144 variants (Table S4) detected via the BSA-seq were submitted to GO enrichment analysis using the GO enrichment analysis database (PlantRegMap; http://plantregmap.gao-lab.org/go.php accessed on 22 May 2022). The candidate genes were classified into two ontological classes: biological processes and molecular functions (Figures S3 and S4). Among the 109 genes, 83 genes had GO annotations, of which 13 terms were significantly (*p* < 0.05) enriched. The GO enriched terms consisted of response to lipid (GO:0033993), response to abscisic acid (GO:0009737), response to alcohol (GO:0097305), response to oxygen-containing compound (GO:1901700), response to acid chemical (GO:0001101), transition metal ion transport (GO:0000041), seed germination (GO:0009845), seedling development (GO:0090351), response to chemical (GO:0042221), cellular response to abscisic acid stimulus (GO:0071215), response to endogenous stimulus (GO:0009719), transition metal ion transmembrane transporter activity (GO:0046915), and double-stranded DNA binding (GO:0003690) (Table 5).

Out of the 13 enriched GO terms, response to lipid (GO:0033993), response to abscisic acid (GO:0009737), transition metal ion transmembrane transporter activity (GO:0046915), and double-stranded DNA binding (GO:0003690) were the most highly enriched (Figures S3 and S4). The annotated genes that were related to the most highly enriched terms consisted of *Glyma.04G199900*, *Glyma.05G243800, Glyma.05G244100, Glyma.06G249800,* and *Glyma.17G219400* (GO:0033993: Response to lipid), and the genes included *Glyma.05G243800*, *Glyma.05G244100*, *Glyma.06G249800*, and *Glyma.17G219400* (GO:0009737: Response to abscisic acid) (Table 5). Two of the annotated genes (*Glyma.05G243800* and *Glyma.05G244100*) were also identified via an SNP/InDel plot and verified by MapChart analysis (Figure 2). The gene *Glyma.05G243800* contained a mutation occurring at the genomic position of 41836669 and encodes a “receptor for activated C kinase 1C” while *Glyma.05G244100* contained mutations occurring at genomic positions of 41855575 (upstream) and 41855657 (upstream), which encodes “phosphatidylethanolamine-binding protein (PEBP) family protein” (Table S5). This result indicates that genes on chromosome 5 within the genomic loci of 41.8–41.9 Mb are putative candidate genes regulating tocopherol content in soybean (Figure 2). Candidate genes associated with transition metal ion transmembrane transporter activity consisted of *Glyma.14G196200* and *Glyma.17G219400*, while *Glyma.01G042900* and *Glyma.08G306100* were associated with double-stranded DNA binding (Table 5). The present study identified new putative genes associated with response to lipid, abscisic acid, transition metal ion transmembrane transporter activity, and double-stranded DNA binding. Thus far, no previous studies have reported a direct or an indirect association of these GO terms with tocopherol expression. Nonetheless, these genes can further be explored for their possible relatedness to tocopherol content in soybean.

## 3. Discussion

### 3.1. Soybean Tocopherol Profiling and WGR Coupled with BSA-Seq

Soybean is a major grain legume and an oilseed crop with an estimated 260 million tons produced globally [33]. The grains contain approximately 20% oil fraction, which is packed with essential compounds, including fatty acids and tocopherols. The grains are an important source of high-quality protein for both humans and animals. As an oilseed crop, the economic value of soybean in the global markets is very much influenced by the rich oil content, fatty acid composition, tocopherol, or VE content. In nutraceutical industries, isoforms of tocopherol are cherished due to their significant health benefits [34,35]. VE is also an important lipophilic antioxidant compound that protects lipoproteins, polyunsaturated fatty acids (PUFA), and intra-cellular membranes from oxidative damage. Consequently, breeding for enhanced tocopherol content is an important objective of soybean.

To identify genomic loci and candidate genes related to tocopherols in soybean seed, the soybean germplasm panel consisting of 1525 accessions (natural population) was cultivated in Beijing (2017 and 2018), Anhui (2017), and Hainan (2017 and 2018) and profiled for seed tocopherol content. The phenotypic data of tocopherol content distribution of the soybean accessions revealed a substantial continuous variation among the accessions (Figure S1). A natural population is composed of a panel of germplasms of different genetic backgrounds, and so constitutes a complete and broad range of variation for diverse traits that can be targeted for genetic studies. Progress in NGS technology has revolutionized various aspects of genomics and thus permitted the ability to rapidly associate molecular markers or genome sequencing polymorphism to phenotypic variation of diverse plant species [36,37]. The Illumina high-throughput sequencing platform is popularly used for constructing double-terminal sequencing libraries to produce a large amount of data in a rapid fashion with a high base accuracy rate that exceeds 98.5% [38]. Likewise, the BSA-seq technique coupled with next-generation sequencing has been frequently used for the rapid identification of several quantitative and qualitative trait loci (QTLs) in many crop species [39,40]. Capitalizing on the phenotypic distribution of tocopherol content among the various accessions, two genomic DNA bulk samples derived from accessions that exhibited contrasting phenotypes for tocopherol were composed for bulked segregant analysis coupled with whole-genome resequencing. Our BSA-seq data also indicate success for the quality and quantity of data obtained, and thus a library construction was achieved.

### 3.2. BSA-Seq Combined with Haplotype Analysis Can Quickly Identify Candidate Genes

In this study, first, we identified 109 candidate genes carrying 144 variant sites related to tocopherols by the BSA-seq method. Then, based on the sliding window analysis, we identified one of the hotspot genomic regions (41.8 and 41.9 Mb) on chromosome 5, which contains six candidate genes (Figure 2). Specifically, one gene *Glyma.05G243400*, encoding translation elongation factor EF1A or initiation factor IF2gamma family protein, was detected, which carries a mutation (T > G) with a genomic variant of Chr05_41807338 that induced a non-synonymous mutation (Table 4). Haplotype analysis was further performed where *Glyma.05G243400* exhibited highly significant variation for tocopherol content across the experimental locations (Figure 4). Results of the haplotype analysis showed that REF coverage was almost 80% for cultivar (pie chart), while in the landrace, the coverage was only about 50% (Figure 4). The current results suggest that cultivars are more reliable resources for the accumulation of tocopherols relative to landraces [41]. Moreover, soybean cultivars are largely developed by targeting high oil content for the oilseed industry by soybean breeders, which indirectly increases the tocopherol content in seeds of soybean cultivars since total tocopherol has a significantly positive correlation with oil [41,42,43]. Thus, breeding for enhanced oil content might have influenced the tocopherol content in the cultivars used in this experiment. Moreover, since soybean cultivars showed significantly higher variation in tocopherol content relative to their landrace counterpart, tocopherol content represents a potential source of new insight into soybean domestication.

### 3.3. Uridine-Diphosphate-Glycosyltransferase (UGT) May Be Involved in Tocopherol Accumulation

We identified a candidate gene, *Glyma.08G184700*, carrying frameshift deletion mutation, which encodes uridine diphosphate (UDP)-glycosyltransferase superfamily protein in chromosome 08 (Table 4). The UGT constitutes a superfamily of enzymes that are involved in the catalysis of glucosidation and facilitates the transfer of glycosyl from UDP-glycosyl donors to diverse lipophilic compounds [44,45]. Members of UGT annotation share a conserved domain of about 50 amino acid residues located in their C-terminal section [45]. The N-terminal of the protein is characterized by sequence variation between isoforms and represents a binding site for diverse lipophilic molecules [46]. Tocopherols are a kind of lipophilic compound or molecule [44,45], but no previous studies have mentioned the role of UGT in tocopherol biosynthesis. However, previous knowledge indicates that the major biological pathways (shikimate, and tocopherol core pathways) through which tocopherol is synthesized are involved in the phenylpropanoid pathway [7,11]. Furthermore, some members of UDP-glycosyltransferase are involved in mediating lignin biosynthesis to flavonoid biosynthesis, which is linked to the phenylpropanoid pathway [47,48]. UDP-glycosyltransferase-related genes (GSA1) have earlier been reported to regulate cell proliferation and expansion under the influence of flavonoid-mediated auxin and related gene expression [49]. This process was found to ultimately influence grain size and enhance abiotic stress tolerance in rice [49]. There are also research findings that reveal that some members of UDP-glucosyltransferase are directly involved in the phenylpropanoid pathway leading to the production of higher levels of secondary metabolites, such as monolignols and flavonoids [50,51]. The first three steps in the phenylpropanoid pathway are catalyzed by phenylalanine ammonia-lyase (PAL), cinnamate 4-hydroxylase (C4H), and 4-coumarate:CoA ligase (4CL), which provide precursors for the downstream metabolic pathway [51]. The shikimate acid pathway is located downstream of PAL, C4H, and 4CL. The association of shikimate acid biosynthesis with the phenylpropanoid pathway suggests a possible role of members of UDP-glucosyltransferase in influencing tocopherol content in plants, though this needs to be functionally dissected in detail.

### 3.4. Lipoxygenases May Be Involved in Tocopherol Accumulation

In this study, we also identified another frameshift deletion candidate gene, *Glyma.15G233300*, encoding lipoxygenases 1 in chromosome 15 (Table 4). Lipoxygenase (LOX) plays a catalytic role in the oxygenation of polyenoic fatty acids, a process that precedes the degradation of storage lipids during seed germination. In soybean, lipoxygenase enzymes, including LOX1, LOX2, and LOX3 [52], are present in mature seeds. They are involved in catalyzing the oxidation of unsaturated fatty acids such as linoleic and linolenic acids to produce conjugated unsaturated fatty acid hydroperoxides, which are then converted to volatile beany off-flavor compounds [52,53]. Previous studies indicated that electron beam irradiation can significantly decrease lipoxygenase activity and simultaneously decline VE activity in soybean seeds [52], which clearly suggests a possible role of lipoxygenases in tocopherol expression. Some members of LOX are known to be expressed in response to phytohormones such as jasmonic acid, salicylic acid, methyl jasmonate, abscisic acid (ABA), and nitric oxide [54,55], which are precursors of the tocopherol biosynthesis pathway, further suggesting a possible role of LOX in tocopherol-related gene expression in soybean. Since different isoforms of LOX might have distinct functional roles, further investigations are needed to validate the candidate genes detected for their possible role in tocopherol biosynthesis.

### 3.5. Tocopherol and Lipid (PUFA) Interaction

Previous studies have revealed a possible biochemical association between tocopherol and lipid oxidation of oils [56]. Thus, genes linked to lipids may play interactive functional roles related to tocopherol content in soybean. PUFAs form part of biological membrane lipids and are often oxidized by ROS because of the presence of multiple double bonds, resulting in lipid radicals [57]. In photosynthetic organisms, tocopherols act as antioxidants for the deactivation of photosynthesis-derived ROS and lipid peroxyl radicals and, consequently, protect membrane lipids from autocatalytic peroxidation [6,35]. Generally, tocopherols are known to interact with membrane lipids to enhance membrane stability [34]. Tocopherols also participate in enhancing seed longevity as well as protecting lipids from oxidation during germination and early seedling growth [58]. This suggests the involvement of tocopherol in lipid metabolism. Thus, in this study, genes (*Glyma.04G199900*, *Glyma.05G243800*, *Glyma.05G244100*, *Glyma.06G249800*, and *Glyma.17G219400*) associated with the GO term “response to lipid” were considered putative genes that express tocopherol in soybean.

### 3.6. Tocopherol and ABA Interaction

Plant-growth regulators or phytohormones are plant signal molecules produced at different stages of plant growth, development, and during plant response to biotic and abiotic stresses [59]. Plants exhibit different forms of biochemical and physiological responses to stress, including the expression of key phytohormones, in particular, abscisic acid, which is expressed under abiotic stress conditions [60]. Tocopherol content increases in seed oil in response to biotic and abiotic stresses [61]. Previous studies suggest the involvement of plant growth regulators or phytohormones in tocopherol synthesis [62,63].

A previous study in rice focused on promoter analyses of the ABA-responsive genes and discovered the presence of several abscisic acid-responsive elements [64]. For instance, an ABA-specific motif has been previously identified in the promoter regions of the VE biosynthesis genes of rice (*OsHPPD*, *OsγTMT*, and *OsMPBQMT1*) [62]. Earlier, Fleta-Soriano and Munné-Bosch [63] also reported a positive correlation between ABA contents and tocochromanol compounds. Additionally, Ghassemian et al. [65] showed that molecular analysis using microarray and gas chromatography-tandem mass spectrometry profiling indicated that ABA-treated Arabidopsis seedlings exhibited increased levels of tocopherol. There was an increased transcriptional level of the tocopherol biosynthesis genes (*HPPD*, *VTE2*, *VTE1*, and *VTE4*) in response to increased ABA treatment [65]. Meaning that the relative expression of *HPPD* (p-hydroxyphenylpyruvate dioxygenase), *VTE2* (Homogentisic acid phytyltransferase encoding homogentisate phytyltransferase), *VTE1* (tocopherol cyclase that converts MPBQ and DMPBQ to δ- and γ-tocopherol, respectively), and *VTE4* (γ-tocopherol methyltransferase: methylates δ- and γ-tocopherol to β- and α-tocopherol, respectively) may be influenced by ABA. The involvement of tocopherols in promoting seed longevity and preventing lipid peroxidation during seed dormancy, seed germination, and early-stage seedling development [58] provides further evidence of existing interactions between tocopherol biosynthesis and ABA signaling. Findings of the previous reports [62,63,65] suggest that the tocopherol biosynthesis might be linked to ABA signaling. In our current study, genes (*Glyma.05G243800*, *Glyma.05G244100*, *Glyma.06G249800*, *and Glyma.17G219400*) associated with the GO term “response to abscisic acid” can be considered as promising candidates associated with tocopherol content in soybean.

## 4. Materials and Methods

### 4.1. Plant Materials and Tocopherol Phenotyping

In this study, a natural population consisting of 1525 soybean accessions collected from various soybean growing regions across the world was used to profile the tocopherol composition of the seeds (Table S3). Accession number, accession name, mean total tocopherol content, accession type, and origin of all accessions are presented in Table S3. Field experiments were carried out in three different locations: Changping, Beijing (40°13′ N and 116°12′ E), Sanya, Hainan (18°24′ N and 109°5′ E) in 2017 and 2018, and Hefei, Anhui (33°61′ N and 117° E) in 2017. The accessions were planted on 12 June 2017 and 14 June 2018 in Changping; 14 November 2017 and 16 November 2018 in Sanya; and on 5 June 2017 in Hefei. Due to a large number of accessions, the accessions were replicated over the locations. At the full maturity stage, each accession was harvested separately, and the grains were used to profile tocopherol composition.

Tocopherol components were detected from matured soybean seeds using a reverse-phase HPLC (Agilent Technologies, Santa Clara, CA, USA) system following the method described by Dwiyanti et al. (2007) [66] with some modifications. The detailed procedure used for tocopherol determination has been previously reported [41]. Tocopherols were quantified using standard curves calculated by the linear regression analysis. The sum of individual tocopherol content was used to compute the total tocopherol content. The phenotypic data based on the tocopherol composition of each accession provided the basis for the selection of individuals to construct a DNA library for bulk sequencing.

### 4.2. Genomic DNA Isolation

At the vegetative stage (V2: one fully expanded trifoliolate leaf presence), leaves were sampled from young, healthy plants of accessions that exhibited extreme contrasting differences for tocopherol composition and used for genomic DNA isolation according to the standard protocol of the Novel Plant Genomic DNA Extraction Kit (DP320, Tiangen Biotech, Beijing, China). The RNase A was used to remove RNA contamination. The concentration and quality of each genomic DNA were measured by the NanoPhotometer^®^ spectrophotometer (Implen, Westlake Village, CA, USA) and 1% agarose gel electrophoresis, respectively. DNA concentration was measured using Qubit^®^ DNA Assay Kit in Qubit^®^ 2.0 Flurometer (Life Technologies, Carlsbad, CA, USA). Only genomic DNA samples with an OD260/280 value ranging from 1.8 to 2.2 were considered good quality DNA for further analyses.

### 4.3. Construction of Genomic DNA Bulks for Sequencing

Two genomic DNA bulks were constructed for bulked segregant analysis sequencing (BSA-seq) based on observed phenotypic data (tocopherol content) of the target population. The two bulk samples for whole-genome resequencing were generated by pooling equal amounts of DNA from a total of 98 soybean germplasms (49 accessions each of high and low tocopherol content) (Figure S1) sampled from the extreme phenotypes. The two DNA bulks were designated as VE-High and VE-Low and submitted to high-throughput sequencing using the Illumina HiSeq™ PE150 platform. Figure S5 illustrates BSA experimental method and data analysis pipeline used. To perform BSA-seq, a total amount of 1.5 μg DNA per sample was used as input material for the DNA sample preparations. The library was constructed according to the protocol of the TruSeqNano DNA HT sample preparation kit (Illumina USA) following the manufacturer’s recommendations, and index codes were added to attribute sequences to different samples. The procedures followed to perform library construction are shown in Figure S6. After the sample was tested, the qualified DNA samples were randomly broken into fragments with a length of 350 bp by a Covaris crusher. The DNA fragments undergo the steps of end repair, adding polyA tails, adding sequencing adapters, purification, and PCR amplification to complete the entire library preparation (Figure S6). The constructed library was sequenced by Illumina HiSeq™ PE150. After the library was constructed, we used Qubit^®^ 2.0 for preliminary quantification, diluted the library to 1 ng/μL, and then used Agilent 2100 Bioanalyzer to detect the insert size of the library. After the insert size was as expected, we used the quantitative polymerase chain reaction (Q-PCR) method to determine the effective concentration of the library. To further confirm the quality of the library, the PCR products were purified (AMPure XP system). An effective library concentration > 2 nM ensures library quality. After the library check was qualified, different libraries were pooled according to the requirements of effective concentration and target data volume, and then the genomic DNA of the two pools was submitted to sequencing using the Illumina HiSeq™ PE150 platform. The sequencing process generated 150 bp paired-end reads with an insert size of 350 bp.

### 4.4. Sequencing Data Quality Analysis

The original image data file obtained from the high-throughput Illumina HighSeq sequencing was analyzed by the CASAVA Base Recognition (Base call) and, subsequently, converted into the sequence reads, also known as raw reads and stored in FASTQ (fq) file format. Reads obtained from the sequencing results, including raw reads that contained connectors or low-quality bases that may affect the subsequent assembly and analyses, were further processed by filtering for quality check. Thus, raw reads were processed by: (1) Removing read pairs containing connectors; (2) Reads that contained ≥ 10% of unidentified nucleotides (N); (3) Reads that contained low-quality (Q ≤ 5) bases that exceeded 50% of the length of the read; (4) Reads with > 10 nt aligned to the adapter, allowing ≤ 10% mismatches; (5) Putative PCR duplicates generated by PCR amplification in the library construction process (read 1 and read 2 of two paired-end reads that were completely identical).

### 4.5. Sequence Alignment

After removing the connectors and low-quality reads, the clean reads obtained were further submitted to quality check to obtain high-quality clean reads. The Burrows–Wheeler Alignment (BWA) algorithm (Version 0.7.10) [67] with the parameter: mem-t 4-k 32-M was used to align and map the resulting high-quality paired-end sequencing reads or clean reads of each sample bulk sequence to the soybean Williams 82 reference genome version 2.0 (Wm82.a2.v1) from Phytozome. The SAMtools software, version 0.1.19 [32], was used to convert alignments to BAM (Binary Alignment/Map) files and for deduplication analysis with the command “rmdup”. Unmapped and non-unique reads were then filtered out. Where multiple read pairs had identical external coordinates, only the pair with the highest mapping quality was retained.

### 4.6. Variant (SNPs and InDels) Detection and Annotation

The Unified Genotyper module of Genome Analysis Toolkit (GATK) software [68], version 3.8 was used for detecting the SNPs and InDels of all DNA samples while the Variant Filtration was used for filtering (SNPs: filtering parameters-cluster Window Size 4, —filter Expression “QD < 4.0||FS > 60.0||MQ < 40.0”, -G_filter “GQ < 20”; InDels: —filter Expression “QD < 4.0||FS > 200.0”, -cluster Window Size 4). The annotation of SNPs and InDels test results was performed using the ANNOVAR software (version 2013Aug23) [69]. ANNOtate VARiation (ANNOVAR) is an efficient software tool for the functional annotation of genetic variants detected by multiple genomes. It gives several pieces of vital information, including chromosomes, gene starting and termination points, reference nucleotides, and mutant nucleotides in which the mutation is located.

### 4.7. Variant (SNP/InDel-Index) Association Analysis

High-quality SNPs and InDels in the bulks were selected for SNP/InDel-index analyses [70]. The SNP- and InDel-index was calculated for all the SNPs and Indels at each SNP/InDel position for the two bulks using the sliding window analysis approach. Here, SNPs/InDels positions that had low-SNP/InDel-index of <0.3 were filtered out. Besides this, prior to association analysis, the SNPs were filtered using the following criteria: SNPs with multiple alleles, SNPs with a sequencing depth of less than 4× in each pool or parent, SNPs with the same genotypes among pools, and SNPs with recessive alleles that were not inherited from parents with recessive genotypes in the pools were all filtered out [71]. Ultimately, a collection of high-quality SNP markers was obtained for use in the association analysis. The SNP- and InDel-index association analysis was used to calculate the genotype frequency difference between the two DNA pools, which is represented as Δ(SNP index) [36,70].

### 4.8. Sliding Window Analysis

A sliding window analysis approach was applied to the SNP- and InDel-index of the whole genome with a 2 Mb window size and 10 Kb step size as default. The average SNP/InDel-index of the SNPs/InDels located in the window was then calculated and used for the sliding window plot. The difference in the SNP/InDel-index of the two bulks was calculated to obtain Δ(SNP/InDel-index) and confidence intervals generated and plotted for all the genomic regions that showed variable read depths. The selected SNPs and InDels detected in the peak regions were annotated and screened for potential functional variants.

### 4.9. Candidate Gene Identification

The SNP/InDel and Δ(SNP/InDel-index) plots were examined in order to identify causal regions. Using the sliding window analysis method, the average SNP/InDel-index and Δ(SNP/InDel-index) were calculated for each genomic interval by using a window size of 2 Mb and step product size of 10 Kb as default. The window graphs were generated and plotted against the genomic positions for the VE-High and VE-Low bulks. The Δ(SNP/InDel-index) of the polymorphic loci between the VE-High and VE-Low pools was then calculated. Only peak regions that were highly consistent across the SNPs and InDels plots were considered to contain candidate genes. Gene models earlier reported in Arabidopsis to be linked to tocopherol biosynthesis were used to predict putative soybean tocopherol candidate genes. The soybean tocopherol genes were subsequently mapped with the annotated candidate genes detected in the BSA-seq. Genes that were found within the genomic loci and matched with the sliding window plots where the peak regions exceeded the threshold value were considered candidate genes. Additionally, genes with non-synonymous mutations were examined for their haplotype structure in our germplasm panel. This consisted of 1525 soybean accessions evaluated in three different locations, including Beijing (2017 and 2018), Anhui (2017), and Hainan (2017 and 2018). Haplotypes were prepared using R statistical software version 4.0.2 (R Foundation for Statistical Computing, Vienna, Austria). The Soybean Functional Genomics and Breeding database (SoyFGB v2.0) (https://sfgb.rmbreeding.cn/, accessed on 18 May 2022) was used for the genotypic data and/or genetic approaches.

### 4.10. GO Analysis of Predicted Candidate Genes

To determine the main biological functions of genes (cellular components, molecular functions, and biological processes), a deep annotation of databases was performed. Based on the 109 predicted candidate genes, the GO analysis was conducted to identify significantly (*p* < 0.05) enriched terms using the PlantRegMap website tool (http://plantregmap.cbi.pku.edu.cn/go_result.php, accessed on 27 May 2022).

## 5. Conclusions

Increased tocopherol composition in soybean has immense potential to enhance the shelf life of seeds, seed vigor, and seed oil quality. Therefore, understanding the genetic mechanisms conditioning tocopherol expression will contribute immensely toward the molecular breeding of soybean with enhanced tocopherol composition in order to fully exploit its diverse benefits. This study was performed to investigate the genomic loci and candidate genes regulating tocopherol content in soybean through a BSA-seq approach. Based on GO annotation analysis, a total of five and four genes were found with their biological functions associated with response to lipid and response to abscisic acid, respectively, which were considered promising candidates associated with tocopherol content in soybean. Following the ANNOVAR analysis, genes containing mutations that occurred in the exonic region, including 10 non-synonymous, 1 stop-gain, and 2 frameshift variants, were preferentially selected as candidate genes related to tocopherol content. Following an SNP/InDel index analysis method, one associated genomic region (from 41.8 to 41.9 Mb) directly corresponding with the sliding window plots where the peak regions exceeded the threshold value was identified on chromosome 5, which contained six putative genes. Among the genes, *Glyma.05G243400* carrying a non-synonymous mutation that encodes a “translation elongation factor EF1A or initiation factor IF2gamma family protein” was identified at the genomic locus of 41.8 Mb. Among the candidate genes, *Glyma.05G243400* was the only gene located in the peak region above the threshold. This gene (*Glyma.05G243400*) was considered the most promising candidate gene related to tocopherol content in soybean. An analysis of haplotype variation also confirmed that *Glyma.05G243400* exhibited highly significant variations in terms of tocopherol content across the experimental locations. Therefore, the results imply the gene *Glyma.05G243400* is the most promising candidate for tocopherol content, which is a novel gene and will serve as a useful gene resource for functional characterization and validation by genome editing. Our present findings hold prospects for molecular marker identification and marker-assisted selection of soybean genetic resources endowed with improved tocopherol content.

## Figures and Tables

**Table 1:** Summary of sequencing data quality.

Sample ^a^	Reference Genome	Number of Plants Bulked	Raw Bases (bp)	Clean Bases (bp)	Effective Rate (%)	Error Rate (%)	Q20 (%)	Q30 (%)	GC Content ^b^ (%)
VE-Low	Williams 82	49	57,765,692,100	57,645,759,600	99.79	0.03	97.66	93.37	36.25
VE-High	Williams 82	49	53,145,146,700	53,070,136,800	99.86	0.03	97.41	92.77	36.52

^a^ VE-Low, bulk DNA pool with low tocopherol content; VE-High, bulk DNA pool with high tocopherol content. ^b^ GC Content stands for guanine-cytosine content.

**Table 2:** Sequencing depth and coverage statistics.

Sample	Mapped Reads	Total Reads	Mapping Rate (%)	Average depth (X)	Coverage at Least 1× (%)	Coverage at Least 4× (%)
VE-High	349,651,428	353,800,912	98.83	41.52	99.61	99.04
VE-Low	379,650,505	384,305,064	98.79	44.57	99.65	99.11

Note: VE-Low, bulk DNA pool with low tocopherol content; VE-High, bulk DNA pool with high tocopherol content.

**Table 3:** Numbers of different types of SNPs and InDels detected between the two soybean bulks.

Category	SNP	InDel
Intergenic	2,701,815	404,485
Upstream	207,958	57,177
Downstream	180,387	44,948
Upstream/Downstream	10,174	2853
Intronic	326,872	69,637
Stop-gain	1614	126
Stop-loss	315	27
Frameshift deletion	-	1902
Frameshift insertion	-	1744
Non-frameshift deletion Exonic	-	1513
Non-frameshift insertion	-	1323
Synonymous	55,156	-
Non-synonymous	74,303	-
Splicing	774	266
Insertion	-	282,838
Deletion	-	304,169
Transitions, conversions	2,325,983	-
Transversions, change	1,233,385	-
Conversion to transversal ratio	1885	-
Total	3,559,368	587,007

Note: Intergenic, variant is in the intergenic region; Upstream, variant overlaps the 1-kb region upstream of the transcription start site; Downstream, variant overlaps the 1-kb region downstream of transcription end site; Upstream/downstream, variant overlaps the 1-kb region upstream of transcription start site for a gene, while it overlaps the 1-kb region downstream of transcription end site of another gene at the same time; Intronic, variant overlaps an intron; Exonic, variant overlaps a coding exon; Stop-gain, an insertion/deletion that leads to the immediate creation of stop codon at the variant site; Stop-loss, an insertion/deletion that leads to the immediate elimination of a stop codon at the variant site; Frameshift deletion, a deletion causing a frameshift; Frameshift insertion, an insertion leading to a frameshift; Non-frameshift deletion, a deletion causing no frameshift; Non-frameshift insertion, an insertion leading to no frameshift; Synonymous, a single-nucleotide variant that does not cause an amino acid change; Non-synonymous, a single-nucleotide variant that does cause an amino acid change; Splicing, variant is within 2 bp of a splicing junction.

**Table 4:** Putative candidate genes containing non-synonymous, stop-gain, and frameshift mutation variants.

Position	Chromosome	Ref	Alt	Effect	Gene ID	Ortholog in Arabidopsis	Description
39758623	Chr05	A	G	Nonsynonymous	Glyma.05G217500	AT1G64450.1	Glycine-rich protein family
41807338	Chr05	T	G	Nonsynonymous	Glyma.05G243400	AT1G18070.2	Translation elongation factor EF1A/initiation factor IF2gamma family protein
48428377	Chr06	C	T	Nonsynonymous	Glyma.06G295300	AT4G28140.1	Integrase-type DNA-binding superfamily protein
17002951	Chr08	A	G	Nonsynonymous	Glyma.08G210000	AT1G15420.1	Repeat-containing protein
14945609	Chr13	A	C	Nonsynonymous	Glyma.13G052400	-	-
46481282	Chr14	C	G	Nonsynonymous	Glyma.14G199800	AT1G13960.1	WRKY DNA-binding protein 4
35333785	Chr16	C	T	Nonsynonymous	Glyma.16G190900	AT2G34930.1	Disease resistance family protein/LRR family protein
36350176	Chr16	C	G	Nonsynonymous	Glyma.16G202100	AT4G05200.1	Cysteine-rich RLK (Receptor-like protein kinase) 25
36970367	Chr16	G	C	Nonsynonymous	Glyma.16G210600	AT5G36930.2	Disease resistance protein (TIR-NBS-LRR class) family
6309627	Chr17	C	T	Nonsynonymous	Glyma.17G081100	AT2G03820.1	Nonsense-mediated mRNA decay NMD3 family protein
6333162	Chr17	A	T	Stop-gain	Glyma.17G081500	-	-
14819413	Chr08	GCAGT	-	Frameshift Deletion	Glyma.08G184700	AT5G24750.1	UDP-Glycosyltransferase superfamily protein
43894622	Chr15	A	-	Frameshift Deletion	Glyma.15G233300	AT1G55020.1	Lipoxygenase 1; Linoleate 9S-lipoxygenase/Linoleate 9-lipoxygenase

Note: Ref, Reference; Alt, Alternative.

**Table 5:** Gene ontology annotations of candidate genes related to tocopherol content in soybean.

GO ID	Term	Annotated	Count	Expected	*p*-Value	Genes
GO:0033993	Response to lipid	565	5	1.02	0.004	Glyma.04G199900, Glyma.05G243800, Glyma.05G244100, Glyma.06G249800, Glyma.17G219400
GO:0009737	Response to abscisic acid	437	4	0.79	0.008	Glyma.05G243800, Glyma.05G244100, Glyma.06G249800, Glyma.17G219400
GO:0097305	Response to alcohol	502	4	0.91	0.013	Glyma.05G243800, Glyma.05G244100, Glyma.06G249800, Glyma.17G219400
GO:1901700	Response to oxygen-containing compound	1104	6	2.00	0.014	Glyma.03G173200, Glyma.04G199900, Glyma.05G243800, Glyma.05G244100, Glyma.06G249800, Glyma.17G219400
GO:0001101	Response to acid chemical	813	5	1.47	0.016	Glyma.04G199900, Glyma.05G243800, Glyma.05G244100, Glyma.06G249800, Glyma.17G219400
GO:0000041	Transition metal ion transport	116	2	0.21	0.019	Glyma.14G196200, Glyma.17G219400
GO:0009845	Seed germination	117	2	0.21	0.019	Glyma.05G243800, Glyma.05G244100
GO:0090351	Seedling development	131	2	0.24	0.024	Glyma.05G243800, Glyma.05G244100
GO:0042221	Response to chemical	2380	9	4.31	0.026	Glyma.03G173200, Glyma.04G199900, Glyma.05G183800, Glyma.05G243800, Glyma.05G244100, Glyma.06G249800, Glyma.08G169800, Glyma.17G219400, Glyma.18G293300
GO:0071215	Cellular response to abscisic acid stimulus	192	2	0.35	0.047	Glyma.05G243800, Glyma.06G249800
GO:0009719	Response to endogenous stimulus	1480	6	2.68	0.050	Glyma.03G173200, Glyma.04G199900, Glyma.05G243800, Glyma.05G244100, Glyma.06G249800, Glyma.17G219400
GO:0046915	Transition metal ion transmembrane transporter activity	103	2	0.21	0.019	Glyma.14G196200, Glyma.17G219400
GO:0003690	Double-stranded DNA binding	150	2	0.31	0.038	Glyma.01G042900, Glyma.08G306100

## Data Availability

Not applicable.

## Supplementary Materials

The following are available online at https://www.mdpi.com/article/10.3390/plants11131703/s1, Figure S1: The frequency distribution of tocopherol content in a natural population of soybean; Figure S2: SNP and InDel distributions between the two soybean sample bulks of tocopherol composition; Figure S3: Gene ontology enrichment analysis of 144/109 genes in the candidate genomic region based on biological function; Figure S4: Gene ontology enrichment analysis based on molecular function; Figure S5: Schematic diagram of the BSA-seq method pipeline; Figure S6: The process of genomic DNA library construction; Table S1: Information about 49 accessions with extremely high tocopherol content; Table S2: Information about 49 accessions with extremely low tocopherol content; Table S3: Information of the 1525 soybean accessions used in this study; Table S4: Information about 144 variants detected in our study via BSA-seq; Table S5: Candidate genes in the QTL region on chromosome 5.
