# Supplementary material for: Identification of Genomic Loci and Candidate Genes Related to Seed Tocopherol Content in Soybean
Source: Plants (Basel). 2022 Jun 27;11(13):1703. doi: 10.3390/plants11131703 (PMC9269242; doi:10.3390/plants11131703)
Supplement: Supplementary file 1 [file plants-11-01703-s001.zip › Supplementary Files/Figure S1-S6.pdf]

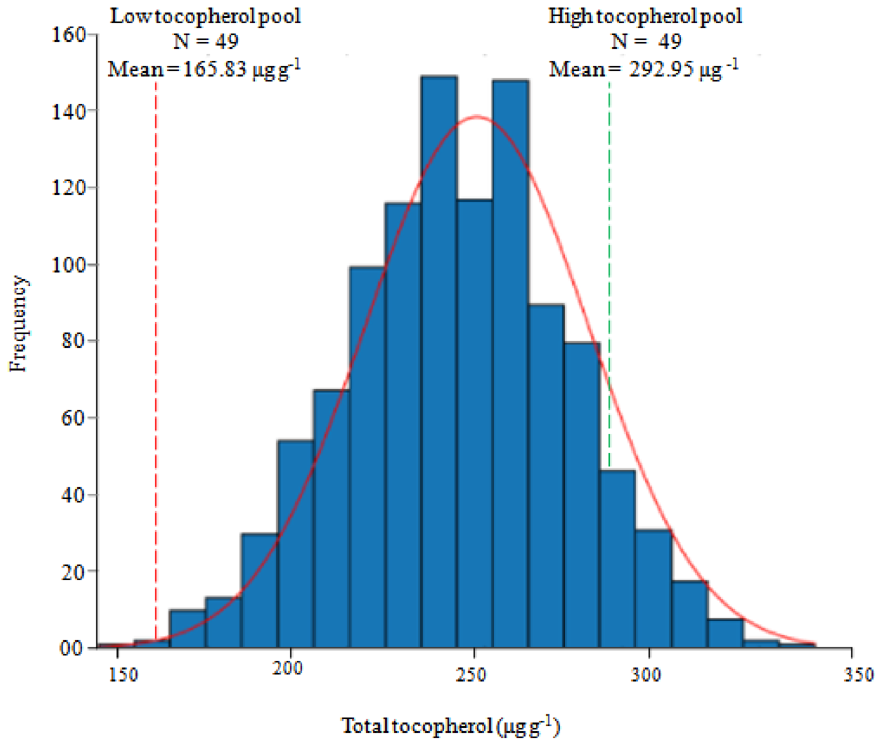

Figure S1. The frequency distribution of tocopherol content in a natural population of soybean. The red and green dashed lines indicate the mean of the low and high tocopherol pools of soybean, respectively.

A

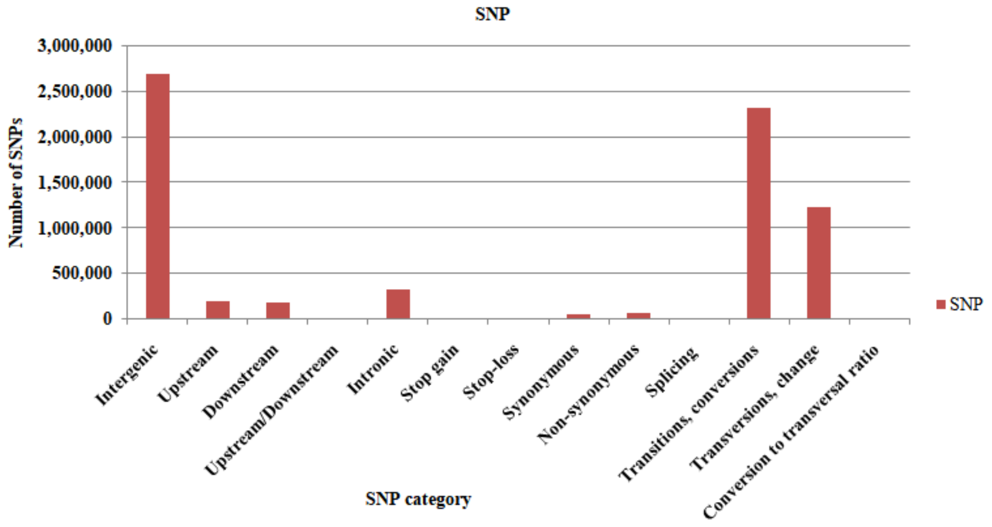

B

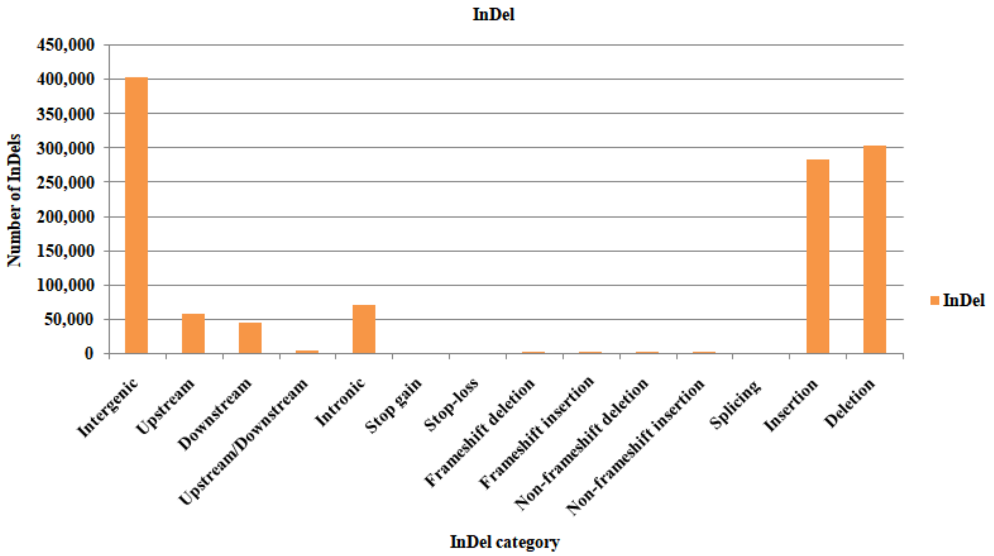

Figure S2. SNPs (A) and InDels (B) distribution between the two soybean sample bulks of tocopherol composition.

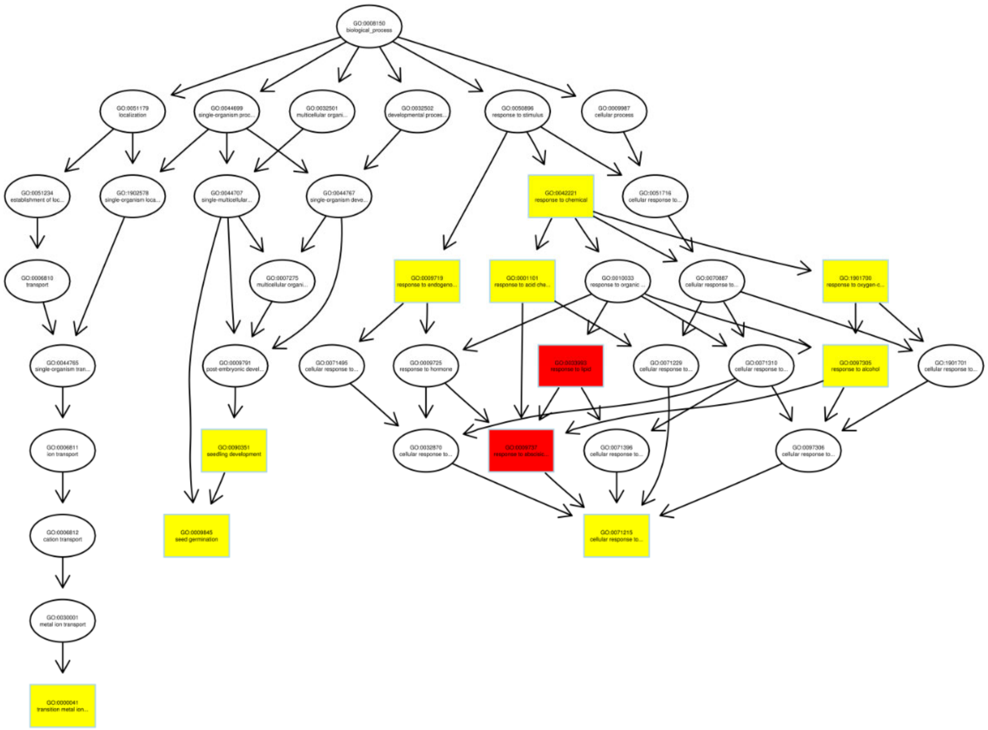

Figure S3. Gene ontology enrichment analysis of 144/109 genes in the candidate genomic region based on biological function. The red boxes represent the two most enriched terms (response to lipid and response to abscisic acid). The yellow boxes are GO enriched terms based on biological function.

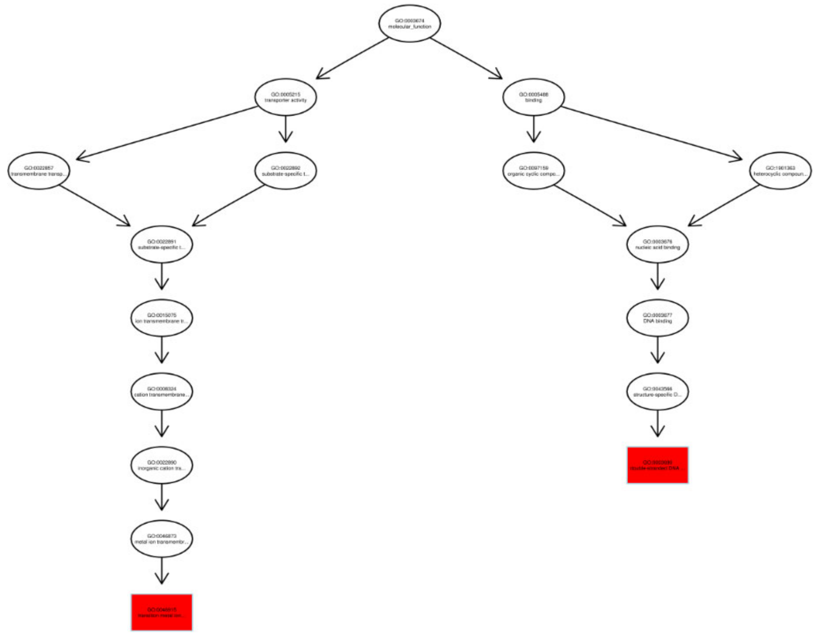

Figure S4. Gene ontology enrichment analysis based on molecular function. The red boxes represent the two most enriched terms (Transition metal ion transmembrane transporter activity and double stranded DNA binding).

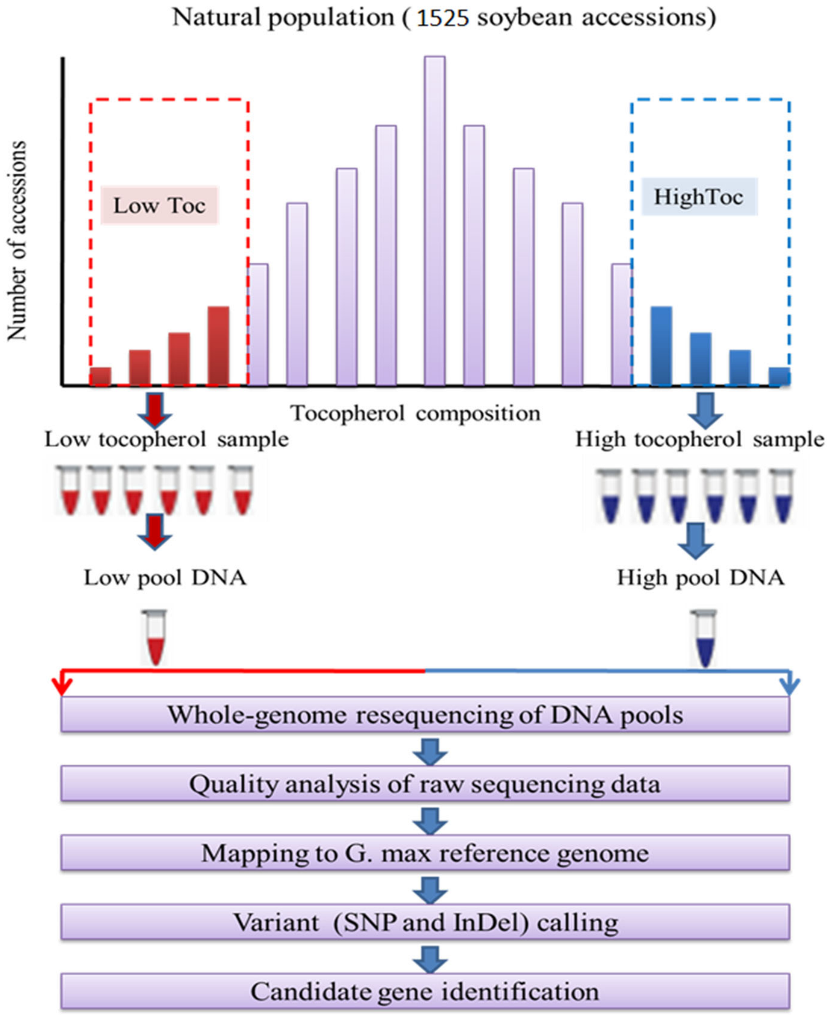

Figure S5. Schematic diagram of BSA-seq method pipeline

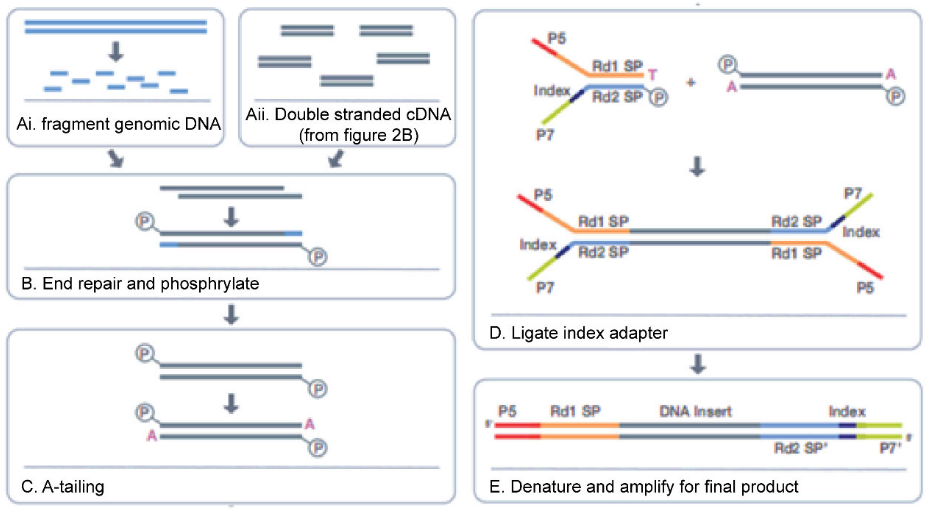

Figure S6. The process of genomic DNA library construction
