# Supplementary material for: Identification of Genomic Loci and Candidate Genes Related to Seed Tocopherol Content in Soybean
Source: Plants (Basel). 2022 Jun 27;11(13):1703. doi: 10.3390/plants11131703 (PMC9269242; doi:10.3390/plants11131703)
Supplement: Supplementary file 1 [file plants-11-01703-s001.zip › Supplementary Files/Table S5.pdf]

Table S5. Candidate genes in the QTL region on chromosome 5

Position	Chromosome	Ref	Alt	Effect	Type	Gene ID	Ortholog in Arabidopsis	Description
41807338	Chr05	T	G	Nonsynonymous	SNP	*Glyma.05G243400*	AT1G18070.2	Translation elongation factor EF1A/initiation factor IF2gamma family protein
41799525	Chr05	T	C	Upstream	SNP	*Glyma.05G243300*	AT1G18060.1	-
41855575	Chr05	A	G	Upstream	SNP	*Glyma.05G244100*	AT1G18100.1	PEBP (phosphatidylethanolamine-binding protein) family protein
41864477	Chr05	G	C	Upstream	SNP	*Glyma.05G244200*	AT3G18050.1	-
41836669	Chr05	T	-	Upstream	InDel	*Glyma.05G243800*	AT3G18130.1	receptor for activated C kinase 1C
41850737	Chr05	-	TTTT	Upstream	InDel	*Glyma.05G244000*	-	-
41855657	Chr05	CA	-	Upstream	InDel	*Glyma.05G244100*	AT1G18100.1	PEBP (phosphatidylethanolamine-binding protein) family protein
